# Lateralization of Executive Function: Working Memory Advantage for Same Hemifield Stimuli in the Monkey

**DOI:** 10.3389/fnins.2017.00532

**Published:** 2017-09-26

**Authors:** Hua Tang, Mitchell R. Riley, Christos Constantinidis

**Affiliations:** ^1^Department of Neurobiology & Anatomy, Wake Forest School of Medicine, Winston-Salem, NC, United States; ^2^School of Life Science and Institute of Life Science, Nanchang University, Nanchang, China

**Keywords:** working memory, monkey, visual field, cerebral hemisphere, visuospatial memory

## Abstract

Working memory capacity, the amount of information that may be maintained in mind over a period of seconds, is extremely limited, to a handful of items. Some evidence exists that the number of visual items that may be maintained in working memory is independent for the two hemifields. To test this idea, we trained monkeys to perform visual working memory tasks that required maintenance in memory of the locations and/or shapes of 3–5 visual stimuli. We then tested whether systematic performance differences were present for stimuli concentrated in the same hemifield, vs. distributed across hemifields. We found little evidence to support the expectation that working memory capacity is independent in the two hemifields. Instead, when an advantage of stimulus arrangement was present, it involved multiple stimuli presented in the same hemifield. This conclusion was consistent across variations of the task, performance levels, and apparent strategies adopted by individual subjects. This result suggests that factors such as grouping that favor processing of stimuli in relative proximity may counteract the benefits of independent processing in the two hemispheres. Our results reveal an important property of working memory and place constraints on models of working memory capacity.

## Introduction

Hemispheric specialization is an important principle of cortical processing, for vision and other sensory modalities (Essen and Zeki, 1978). The primary visual cortex of vertebrates displays hemispheric specialization so that information from one hemifield is represented by neurons located in the contralateral hemisphere. This contralateral bias is diminished in successive stages of processing along the cortical pathways, and neurons with bilateral or ipsilateral receptive fields are more frequent in the posterior parietal and inferior temporal cortices (Mountcastle et al., 1975; Desimone and Gross, 1979). Nonetheless, a number of psychophysical phenomena reveal perceptual advantages for stimuli presented in the same hemifield. For example, subjects are faster at detecting repeating visual stimuli, perceiving illusory contours, or judging whether stimuli are the same or different when presented in the same hemifield (Pillow and Rubin, 2002; Butcher and Cavanagh, 2008; Hayes et al., 2010).

Among higher cognitive functions, language and visual-spatial representations are strongly lateralized in humans, so that their processing occurs predominantly by a single hemisphere (Corballis, 2012; Skeide and Friederici, 2016). Executive functions, including working memory, are not thought to be lateralized, nor has any advantage been associated with processing involving a single hemisphere. To the contrary, a bilateral field advantage has been postulated for tasks with high computational complexity, which might benefit from the processing power of two hemispheres (Leblanc-Sirois and Braun, 2014: Umemoto et al., 2010). Working memory and attention are notoriously subject to a processing bottleneck which limits how many items can be processed or maintained in mind at any point in time (Constantinidis and Klingberg, 2016). Splitting items between the left and right hemifield has been shown to confer an advantage when maintaining in memory multiple items, as parallel processing by the left and right hemisphere can expand the capacity of visual information processing (Delvenne, 2005; Delvenne et al., 2011; Hudson et al., 2012). In recent years, direct evidence has emerged that working memory capacity and spatial attention may operate independently in the two hemifields (Alvarez and Cavanagh, 2005; Alvarez et al., 2012; Stormer et al., 2014).

On the other hand, factors, such as grouping, Gestalt principles of proximity and connectedness, and the specific arrangement of stimuli may confer an advantage for groups of stimuli maintained in memory, over stimuli distributed between fields (Jiang et al., 2000; Xu, 2006; Peterson and Berryhill, 2013). It is not obvious, therefore, that multi-stimulus displays will provide a general advantage when distributed between hemifields over being concentrated in the same field. Within-field advantages are thought to occur due to processes, such as perceptual grouping, which likely originate in early visual cortex and are propagated along the visual pathways (Banich, 1998a,b; Weissman et al., 2000).

Non-human primates are capable of mastering tasks requiring memory for multiple stimuli (Buschman et al., 2011; Heyselaar et al., 2011; Lara and Wallis, 2012), which allows for the neural basis of working memory capacity and its limitations to be investigated with neurophysiological means. Indeed, neurophysiological studies have begun to investigate the representation of multiple-stimulus information in memory by individual neurons (Warden and Miller, 2007; Buschman et al., 2011; Lara and Wallis, 2012). Experiments in monkeys also suggest that activity representing multiple items in memory declines to a greater extent when these appear in the same hemifield as opposed to different hemifields, thus eroding the information representing the multiple items, in each hemifield (Buschman et al., 2011; Matsushima and Tanaka, 2014). However, monkeys may employ different strategies to perform multi-item working memory tasks (Wittig et al., 2016).

We were motivated to investigate how general such bilateral benefits are across working memory tasks in non-human primates, and across individual subjects. We therefore trained monkeys in variations of tasks that require memory for multiple stimuli and determined their behavior for displays containing stimuli in one or both hemifields.

## Materials and methods

Four male, rhesus monkeys (*Macaca mulatta*) weighing 7–13 kg were used in this study. Experiments were carried out in accordance with the recommendations of the U.S. Public Health Service Policy on Humane Care and Use of Laboratory Animals and the National Research Council's *Guide for the Care and Use of Laboratory Animals*. The protocol was approved by the Wake Forest University Institutional Animal Care and Use Committee.

### Behavioral tasks

The monkeys faced a computer monitor 60 cm away in a dark room with their head fixed, as described in detail previously (Qi et al., 2010; Meyer et al., 2011). Eye position was sampled at 240 Hz, digitized, and recorded with an infrared eye position tracking system (model RK-716; ISCAN, Burlington, MA). The visual stimulus presentation and behavior monitoring were controlled by in-house software (Meyer and Constantinidis, 2005) implemented in the MATLAB computational environment (Mathworks, Natick, MA), using the Psychophysics Toolbox (Brainard, 1997). Monkeys were required to maintain their gaze on the fixation target throughout a trial; breaks in fixation aborted the trial.

**Figure 1:**
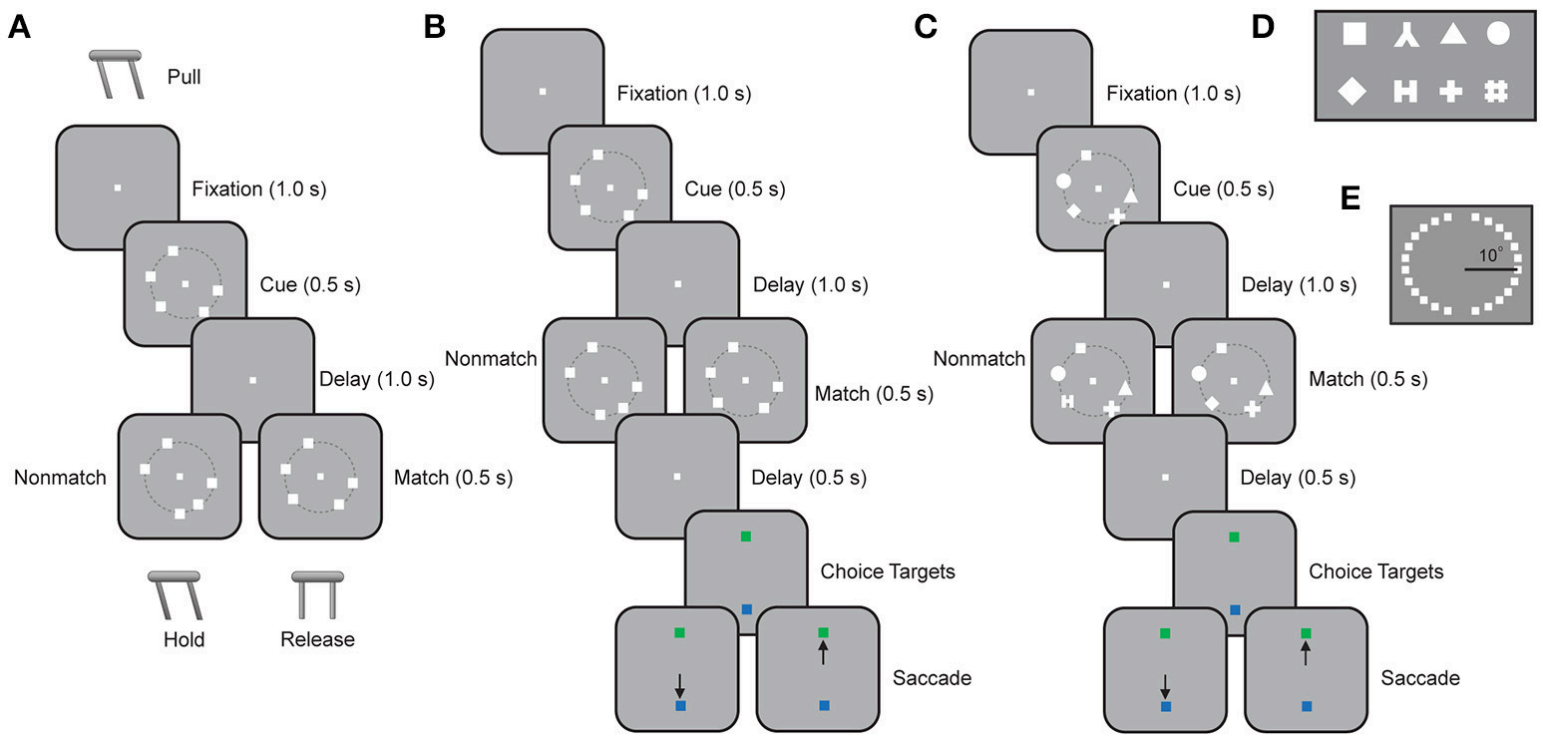
Working memory capacity tasks. **(A)** Successive frames illustrate the sequence of stimulus presentations in the spatial Match/Nonmatch task requiring a lever pull. The cue is presented and after a delay period, a match or nonmatch stimulus display appears. The monkeys were required to remember the locations of all the squares in the cue stimulus and to withhold the lever if an identical stimulus display appeared (match). If one of the squares appeared at a new location, then the display constituted a nonmatch and the monkey was required to release the lever. **(B)** Schematic illustration of the spatial Match/Nonmatch task requiring an eye movement to one of two choice targets. The sequence of displays is the same as in **(A)**, but two choice targets were presented at the end of a trial. The monkey was required to saccade to a green target if the two sequential displays matched each other, or to a blue target otherwise. **(C)** Schematic illustration of the feature Match/Nonmatch task. The monkey was now required to remember the shapes of stimuli appearing at different locations during the cue interval. During the second stimulus presentation, an identical display constituted a match, and the monkey was required to saccade to a green target. Alternatively, the same number of stimuli appeared, at the same locations, but one stimulus differed in shape from the cue display, and constituted a nonmatch. The monkey was then required to saccade to the blue target. **(D)** Stimulus set consisting of eight possible shapes in the feature Match/Nonmatch task. **(E)** The 24 possible locations where stimuli could appear in the spatial Match/Nonmatch task.

All four monkeys were trained with spatial versions of the Match/Nonmatch task (Figures 1A,B). Additionally, one of the monkeys was trained with a feature Match/Nonmatch task (Figure 1C). The basic spatial Match/Nonmatch task (Figure 1A) required monkeys to remember the locations of multiple stimuli in a cue display and to determine if a second display was identical or not. For two monkeys (DA and CA) the trial started with the monkeys pulling the lever to initiate the trial, and keeping their eyes fixated on a central fixation target. After 1 s of stable fixation, a cue display was presented for 0.5 s. The cue display consisted of 1–5 white squares, measuring 1.5° of visual angle in size. Each square was displayed at 1 of 24 possible locations arranged on a circle at an eccentricity of 10° of visual angle, with a 15° angular separation between locations (Figure 1E). This was followed by a delay period of 1 s when only the fixation target was visible. Subsequently, a second display appeared with the same number of stimuli as the cue, either at identical locations (constituting a match), or with one item appearing at a different location (constituting a nonmatch). The second display was presented for 0.5 s and after its offset the animals were required to release the lever within 0.5 s if it was a match, or to continue holding for 0.5 s if it was a nonmatch. The monkeys received a liquid reward for a correct response. The trial was immediately aborted if the monkeys released the lever at any other time during the trial, or if the monkeys broke fixation at any point prior to the lever release.

The other two monkeys (EL and NI) were trained in a variation of this spatial Match/Nonmatch task (Figure 1B). For these animals, a trial started with a 1.0 s fixation interval. Then a cue display was presented for 0.5 s, containing 1–5 white squares, as in the basic task. After a delay period of 1.0 s, a second display appeared with the same number of stimuli. Two choice targets appeared subsequently at the bottom and top of the screen, one of which was green and the second blue, with their position pseudo-randomly interleaved between trials. The monkey was required to saccade to the green target if the two stimuli displays were identical, and to the blue target if they were not.

One monkey was additionally trained in the shape Match/Nonmatch task (Figure 1C). In this task, the monkey was required to remember the shapes of stimuli appearing at multiple locations. The trial started with a 1.0 s fixation interval and then a cue display was presented for 0.5 s. The cue display comprised 1–5 white geometric shapes fitting within a 2° aperture, drawn from a set of 8 shapes (Figure 1D), which we have described in detail before (Meyer et al., 2007). These were displayed at 1 of 16 locations arranged on a circle at an eccentricity of 10° of visual angle, with 22.5° angular separation between locations. After a delay period of 1 s, a second display appeared with the same number of stimuli at identical locations. In the second display, either all shapes were identical, or one new shape substituted one of the shapes in the cue display. The monkey was required to saccade to the green choice target appearing at the end of the trial if the two stimuli displays were identical, and to the blue target if they were not.

### Data analysis

Behavioral performance in the Match/Nonmatch task was determined in daily sessions, based on the proportion of correct responses for each stimulus display. Trials that were prematurely terminated, e.g. because of a break in fixation, or due to release of the lever before the match/nonmatch stimulus was even displayed, were omitted from this analysis. Performance reflects only the proportion of correct and incorrect choices in completed trials. Displays were grouped based on the number of stimuli, and on the arrangement of stimuli in the same hemifield, or across hemifields. Analysis of performance levels across displays of different numbers of stimuli was performed with non-parametric 1-way tests, the rank-sum (also known as Mann-Whitney) test, equivalent to the parametric *t*-test, and the Kruskal-Wallis test, equivalent to the one-way ANOVA test, and the non-parametric two-way Scheirer-Ray-Hare test, equivalent to the two-way ANOVA test. In all instances, the proportion of correct trials in one behavioral session (typically of 150–200 correct trials) was used as one observation. A regression model was also used, testing the dependence of performance on the number of stimuli in the display, the mean distance between stimuli, and whether they all appeared in the same side or not. The distance used for this analysis was defined as the mean distance of all possible stimulus combinations in the display. All analysis of behavioral data was performed in the MATLAB environment, version R2012-2015a (Mathworks, Natick MA).

## Results

**Figure 2:**
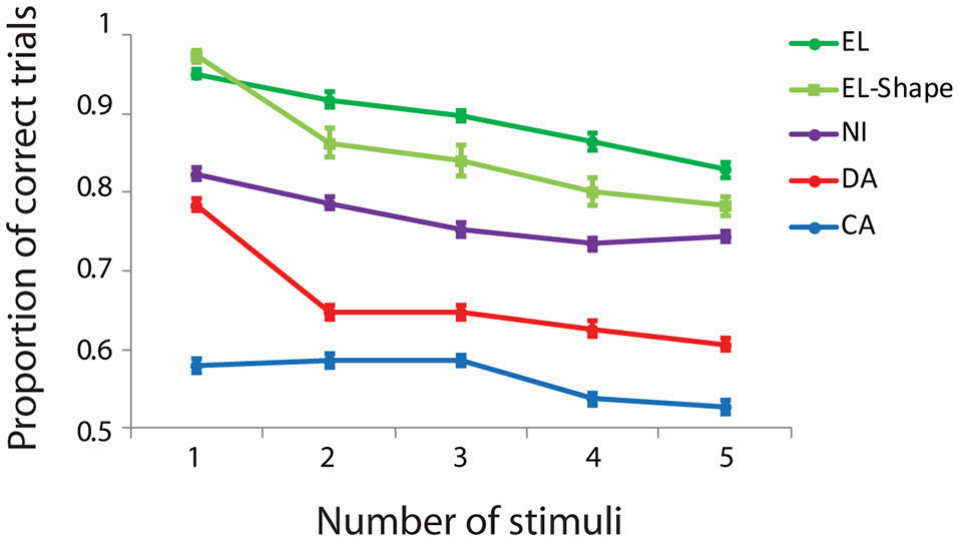
Behavioral performance in the working memory capacity task as a function of number of stimuli. Proportion of correct trials is plotted for four different subjects (EL, NI, DA, and CA) in the spatial task, and one subject (EL) in the shape task. Error bars represent mean ± SEM across daily sessions.

We trained monkeys to perform working memory tasks that required memory of multiple visual items. In a spatial Match/Nonmatch task (Figures 1A,B), four monkeys viewed a sample display with 1–5 white squares. After a delay period of 1 s, a second display appeared with the same number of stimuli, either at identical locations, or with one item appearing at a different location. The subjects were required to judge whether the two stimulus displays were the same or not. We recorded performance in the task from four monkeys at different stages of training, achieving different overall levels of performance in the task (Figure 2). Generally, performance decreased as a function of number of stimuli that the monkeys had to remember. Across all conditions and numbers of stimuli, subject EL had an overall correct performance of 89% (*n* = 115 sessions, 24,317 total trials). Subject NI achieved an overall performance of 79% (*n* = 84 sessions, 19,835 trials). Subject DA achieved an overall performance of 66% correct (*n* = 96 sessions, 21,998 total trials). Subject CA's overall performance was 56% (*n* = 55 sessions, 23,061 total trials).

### Same hemifield vs. different hemifields

To determine if capacity was independent in the left and right hemifields, we analyzed performance for stimulus displays divided into different groups depending on the number of stimuli appearing in different hemifields. Performance generally declined as a function of stimulus number (Figure 2). We examined displays with at least 3 or more stimuli, for which sufficient numbers of errors were available from all monkeys. We then examined the spatial determinants that influenced performance across all possible 3-, 4-, and 5-stimulus configurations. Displays that contained exactly 3 stimuli were grouped into two groups termed “3+0” and “2+1” depending on whether all 3 stimuli appeared in one hemifield, or if 2 stimuli appeared in one hemifield, and 1 stimulus in the other. For 4-stimulus displays three groupings were possible: “4+0,” “3+1,” and “2+2.” For 5-stimulus displays also three groupings were possible: “5+0”, “4+1,” and “3+2.” As a control, we analyzed performance for stimulus displays in the upper and lower hemifield. Stimulus displays could be grouped in the exact same fashion for upper- and lower-field stimuli, as well.

**Figure 3:**
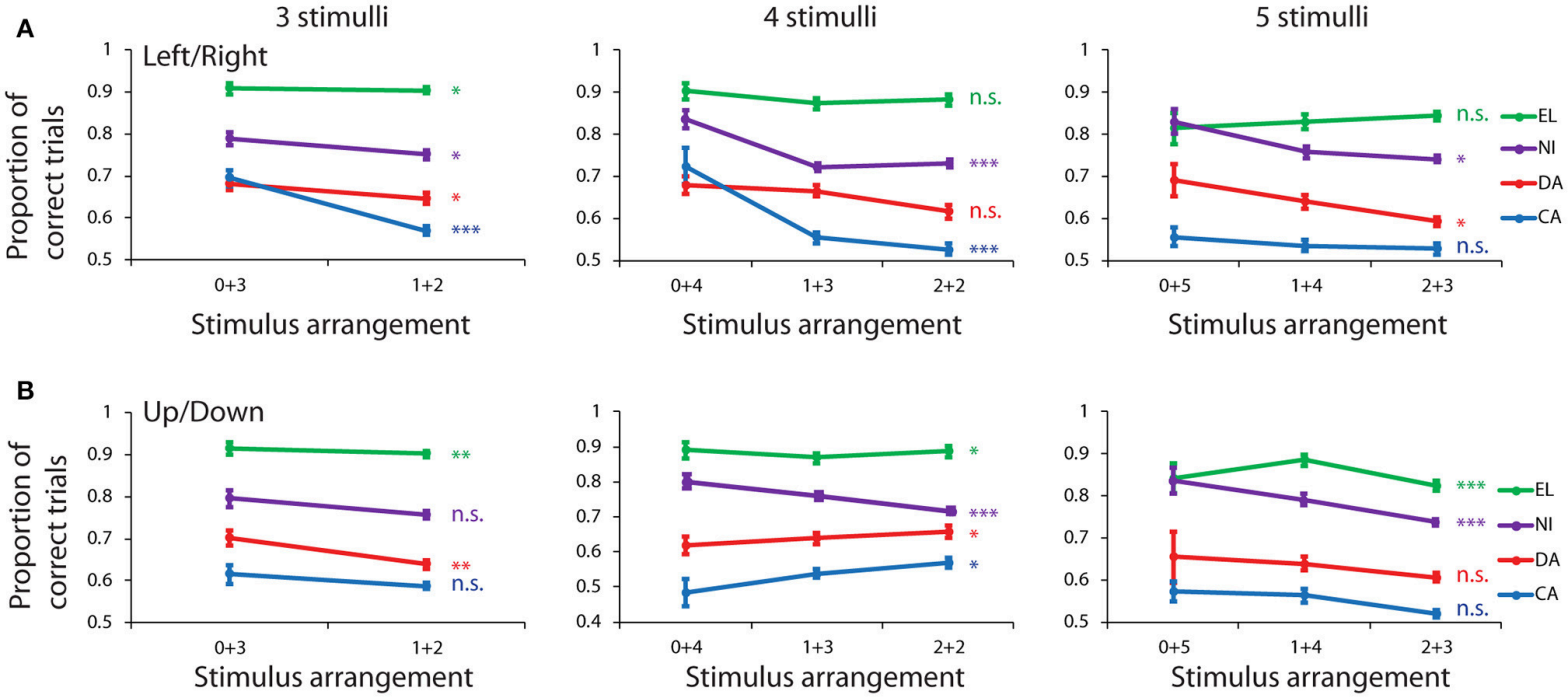
Behavioral performance in the spatial Match/Nonmatch task for different stimulus groups. **(A)** Proportion of trials that ended in a correct response is shown for different numbers of stimuli appearing in the left and right hemifield. Data from four subjects are shown in different colors: EL, NI, DA, and CA. **(B)** Stimulus groups were sorted between up and down hemifields. Error bars represent mean ± SEM. ^*^p < 0.05, ^**^p < 0.01, ^***^p < 0.001, n.s., not significant.

Contrary to what we would expect if working memory capacity saturated independently in the two hemifields, when the stimulus groups were defined based on arrangement of stimuli on the left and right field, performance was poorer when stimuli appeared in both hemifields, than when they appeared in the same hemifield (Figure 3A). For displays with 3 stimuli, a significant difference was present for all four animals: subject CA (rank-sum test, *p* = 1.4 × 10^−8^, *n* = 55 sessions with 3+0 stimuli, 55 sessions with 2+1 stimuli), DA (rank-sum test, *p* = 0.045, *n* = 95 sessions with 3+0 stimuli, 96 sessions with 2+1 stimuli), NI (rank-sum test, *p* = 0.019, *n* = 72 sessions with 3+0 stimuli, 84 sessions with 2+1 stimuli), EL (rank-sum test, *p* = 0.041, *n* = 93 sessions with 3+0 stimuli, 115 sessions with 2+1 stimuli). The improvement represented a 12.5% increase in total performance for subject CA who achieved the lowest overall performance, a 3.9% increase for subject NI, a 3.5% increase for subject DA, and a 0.5% improvement for subject EL, who achieved > 90% performance for this type of stimuli.

For displays with 4 stimuli, again the lowest performance was observed when stimuli appeared in both fields, than when they all appeared either in the left or right side. The effect reached statistical significance, evaluated with a Kruskal-Wallis test, for two animals CA (*H* = 17.33, *df* = 2, *p* = 1 × 10^−4^, *n* = 10 vs. 55 vs. 55 sessions for the three possible arrangements) and NI (*H* = 23.75, *df* = 2, *p* = 7 × 10^−6^, *n* = 54 vs. 84 vs. 84 sessions). The benefit represented an 18.4 and 11.0% improvement for stimuli in the same field, over the average of the two split-stimulus arrangements. A similar trend, albeit without reaching significance, was present for monkey DA (3.7% improvement, *p* = 0.063) and EL (1.7% improvement, *p* = 0.069).

For displays with 5 stimuli a significantly different performance was also seen in two monkeys, with performance being lower when stimuli appeared in both hemifields than all in the same hemifield: subject DA (Kruskal-Wallis test, *H* = 8.25, *df* = 2, *p* = 0.016, *n* = 22 vs. 96 vs. 96 sessions) and subject NI (Kruskal-Wallis test, *H* = 8.65, *df* = 2, *p* = 0.013, *n* = 29 vs. 83 vs. 84 sessions). This improvement represented a 7.5 and 8.2% advantage in performance for stimuli in the same field. A similar trend was observed in monkey CA with a 2.4% improvement for unilateral stimuli (*p* > 0.3). The only exception across all conditions was monkey EL, which exhibited a trend in the opposite direction, of a 2.4% benefit in performance when 5 stimuli were split between two hemifields. However, the effect size was minimal (Cohen's *d* = 0.15 for one-side vs. split displays pooled together) and the difference did not reach statistical significance (*p* > 0.8). In summary, the greatest performance advantages were observed when all stimuli appeared in the same hemifield, and this advantage was generally greater in subjects performing the task at lower levels.

### Upper vs. lower visual field

Displays were also more difficult to remember when stimuli were split between the upper and lower hemifield, than when they appeared in only one of the two (Figure 3B). The effect reached statistical significance for subject DA in displays with 3 stimuli (rank-sum test, *p* = 0.006, *n* = 83 vs. 96 sessions), for subject NI in displays with 4 and 5 stimuli (4 stimuli: Kruskal-Wallis test, *H* = 14.87, *df* = 2, *p* = 6 × 10^−4^, *n* = 36 vs. 83 vs. 83 sessions; 5 stimuli: Kruskal-Wallis test, *H* = 16.82, *df* = 2, *p* = 2 × 10^−4^, *n* = 15 vs. 77 vs. 84 sessions) and for subject EL in displays with 3, 4, and 5 stimuli (3 stimuli: rank-sum test, *p* = 0.001, *n* = 91 vs. 115 sessions; 4 stimuli: Kruskal-Wallis test, *H* = 7.5, *df* = 2, *p* = 0.024, *n* = 49 vs. 112 vs. 114 sessions; 5 stimuli: Kruskal-Wallis test, *H* = 17.9, *df* = 2, *p* = 1 × 10^−4^, *n* = 28 vs. 83 vs. 115 sessions). The only exceptions were subjects CA (Kruskal-Wallis test, *H* = 7.65, *df* = 2, *p* = 0.022, *n* = 10 vs. 55 vs. 55 sessions) and DA (Kruskal-Wallis test, *H* = 6.9, *df* = 2, *p* = 0.032, *n* = 46 vs. 96 vs. 96 sessions) in displays involving 4 stimuli, in which case, slightly lower performance was observed when all stimuli appeared in either the lower or upper field.

The results of this analysis indicate that dividing the hemifield in up and down halves generally produced performance advantages for stimuli appearing in the same hemifield, though this effect was less consistent than dividing into left and right fields. This result confirms that potential benefits from independent processing of stimuli in two fields was rare, and in those occasions that an advantage was observed, it could not be attributed to independent left vs. right hemisphere processing, but rather appeared for stimuli distributed between the upper and lower field (Figure 3B, middle).

### Left vs. right visual field

We also examined whether there was an overall advantage for stimuli appearing specifically in the left or in the right hemisphere. The results reported above regarding unilateral displays were averaged from the left and right hemifields, and might have obscured an improved performance in one, concurrent with a diminished performance in the other field. Data from three animals were sufficient to perform comparisons for displays with 3–5 stimuli, all appearing on the same side (**Figures 5A–C**). A Scheirer-Ray-Hare test with factors left/right side, and number of stimuli revealed no significant main effect for side in any of the subjects: subject EL (*H* = 2.804, *df* = 1, *p* > 0.05); subject NI (*H* = 1.501, *df* = 1, *p* > 0.05); subject DA (*H* = 2.424, *df* = 1, *p* > 0.1). The result confirmed our expectation that working memory capacity is not strongly lateralized, so as to confer an advantage for stimuli appearing in one of the two hemifields.

### Stimulus distance

The similar results observed for left/right and upper/lower fields led us to suspect that the distance between stimuli may be the most important factor that determined the variability of behavioral performance across stimulus displays, rather than the specific arrangement of stimuli in these hemifields. To test the effect of these variables we created a regression model that incorporated number of stimuli, average distance between stimuli and left/right side as independent variables:

(1) $$P=\beta_{0}+\beta_{1}NUM+\beta_{2}DIST+\beta_{3}SIDE+\varepsilon$$

Here *P* represents performance (percent correct responses in a session), *NUM* the total number of stimuli in the display, *DIST* the mean distance between stimuli calculated by averaging all possible angular distances between stimuli in the display, and *SIDE* a binary variable representing displays with stimuli either split between fields, or all appearing in the same (either left or right) field.

As expected, the coefficient corresponding to stimulus number was negative (i.e., higher performance was observed for displays with fewer stimuli), and highly significant for all monkeys (regression analysis, subject EL: *p* = 7.9 × 10^−22^, subject NI: *p* = 0.0197; subject DA: *p* = 0.0011, subject CA: *p* = 1.98 × 10^−9^). The effects of distance and side did not prove to be uniform across monkeys, however. For subject EL, we observed a significant positive distance coefficient (regression analysis, *p* = 0.015) suggesting that this monkey benefitted from stimuli spread out. Once the distance variable was included in the model, however, no significant effect of whether the stimuli were concentrated or split between hemifields was present (regression analysis, *p* = 0.34). In other words, as long as the distance between stimuli was large, there was no further benefit for stimuli appearing in different hemifields. In contrast, monkeys NI and CA exhibited a significantly negative distance coefficient (regression analysis, *p* = 0.002 and *p* = 1.22 × 10^−5^, respectively) suggesting that for these animals, tighter grouping of stimuli offered an advantage. A significant benefit of side was also present (*p* = 0.001 and *p* = 0.0057, respectively), in the direction of higher performance for stimuli in the same hemifield. Finally, subject DA exhibited a significantly negative distance coefficient (regression analysis, *p* = 0.02), suggesting that for this animal too, tighter grouping of stimuli offered an advantage, though, once distance was accounted for, no significant effect of whether the stimuli were concentrated or split between hemifields was present (*p* = 0.54).

These results reveal a considerable variability in strategies adopted by monkeys and stimulus arrangements that are easier to recall by each. In no instance however, independent hemifield processing accounted for an improvement in performance.

### Shape working memory task

The results discussed so far were obtained from a task that only required memory for the spatial location of the stimuli. We considered that this effect may lend itself to grouping of stimuli into patterns or other type of mental transformation of the stimulus display. Therefore, we trained one monkey to perform a working memory task requiring memory of both the location and shape of the stimuli (Figure 1C). The monkey now had to observe a display of different stimuli, drawn from a set of eight white geometric shapes (Figure 1D). After a delay period, a second display was shown and the monkey had to determine if any of the shapes was different or if the second display was identical to the first. Subject EL achieved an overall performance of 85% correct trials (Figure 2) in this task (*n* = 34 sessions, 8,425 total trials).

**Figure 4:**
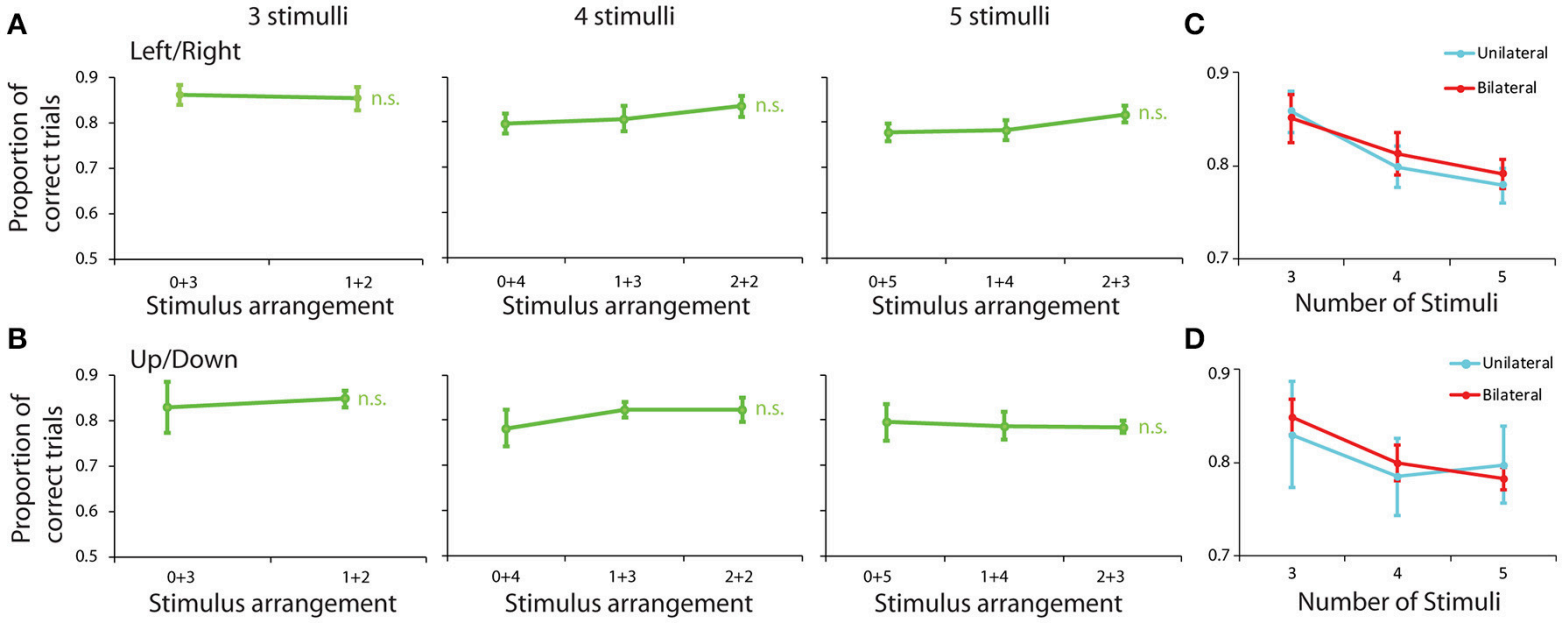
Behavioral performance in the feature Match/Nonmatch task for different stimulus groups. **(A)** Proportion of correct trials are shown for stimulus groups sorted between left/right hemispheres. **(B)** Stimulus groups were sorted between up/down hemispheres. Error bars represent mean ± SEM, n.s. indicates not significant. **(C)** Proportion of correct trials depicted in **(A)**, now averaged across all bilateral and unilateral displays in the left and right field, and plotted as a function of number of stimuli. **(D)** Proportion of correct trials depicted in **(B)**, now averaged across all displays in the upper and lower field, and plotted a function of number of stimuli.

**Figure 5:**
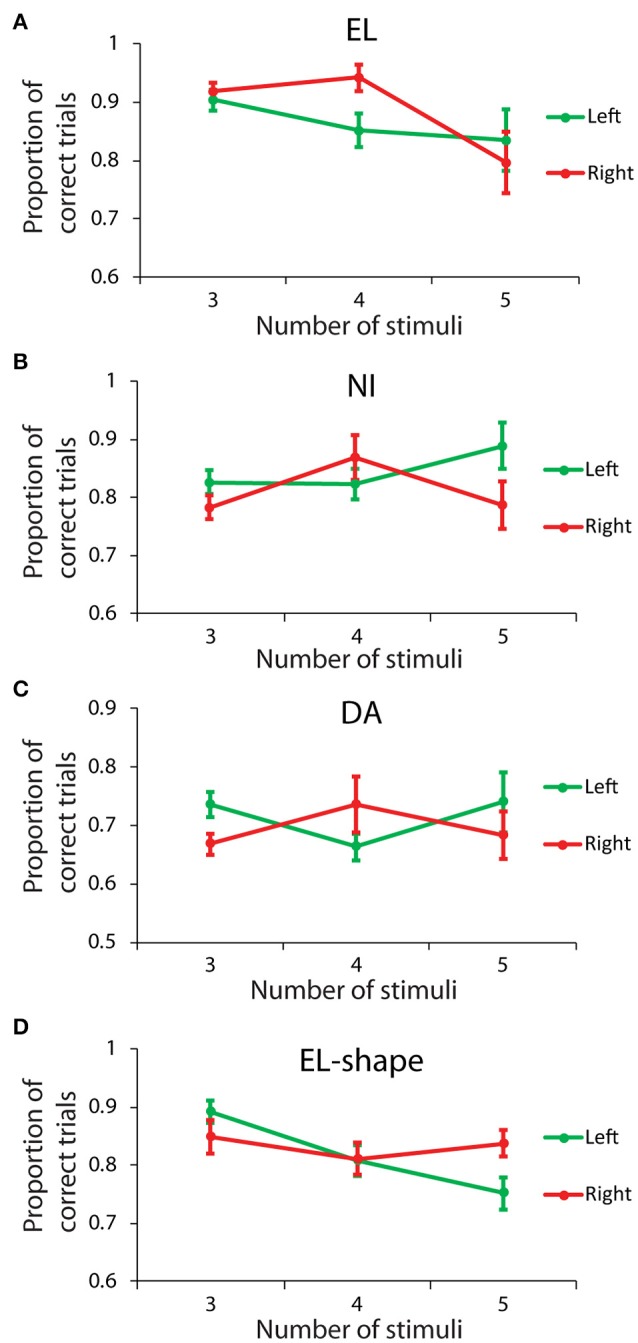
Behavioral performance for stimuli appearing either in the left or the right hemisphere. **(A–C)** Proportion of correct trials from three monkeys (EL, NI, DA), in the spatial Match/Nonmatch task, for different numbers of stimuli, all displayed in either the left (green) or right (red) hemifields. **(D)** Proportion of correct trials for monkey EL in the shape task.

We repeated the analysis of performance based on arrangement of stimuli across hemifields, using the same groups of displays as in the spatial task. There were no significant differences for groups of stimulus displays, based on their appearance of either the left vs. right or the upper vs. lower hemifield (Figures 4A,B). To ensure that we had sufficient power to detect a potential difference between conditions in this experiment, we pooled performance from all possible bilateral displays (e.g., 1+3 and 2+2 arrangement in displays with 4 stimuli) and we performed a Scheirer-Ray-Hare test using the number of stimuli and the unilateral or bilateral arrangement of stimuli as factors (Figures 4C,D). When examining performance in left/right hemifields, we detected a significant effect of stimulus number (*H* = 9.097, *df* = 2, *p* = 1.7 × 10^−4^), but again no significant effect of unilateral/bilateral arrangement (*H* = 0.181, *df* = 1, *p* > 0.6). Still, we did observe an overall trend toward higher performance for displays where all stimuli were distributed between the two fields vs. present in the same field. The average difference between these two conditions was 0.7% (81.2 and 81.9%, respectively). This advantage, however, was no greater than the difference in performance for stimuli appearing all in the upper or lower hemifields (79.7%) vs. distributed between upper and lower field (81.0%), which also did not reach significant difference (Scheirer-Ray-Hare test, *H* = 0.369, *df* = 1, *p* > 0.5 for effect of unilateral/bilateral displays in Figure 4D). There was no significant effect of having all stimuli specifically in the left vs. the right side of the screen in this task, either (Scheirer-Ray-Hare test, *H* = 0.501, *df* = 1, *p* > 0.4 in Figure 5D).

We also repeated the regression analysis described above for the analysis of performance in the shape working memory task. The results revealed similar preferences that monkey EL displayed for the spatial task as well. A slightly negative coefficient was present for the distance variable, suggesting a small preference for spread out displays, however this effect failed to reach significance for the shape task (regression analysis, *p* = 0.085). Once the distance variable was included in the model, the coefficient representing whether the stimuli were concentrated or split between hemifields was also not significant (regression analysis, *p* = 0.1). In this case too, we conclude that working memory capacity does not improve when stimuli are distributed between the left and right side of the field, as would be predicted if two independent capacities operated in the two cerebral hemispheres.

## Discussion

Our study set out to test if working memory performance for displays of multiple stimuli is higher when stimuli are distributed across the left and right hemifield, as might be predicted if this ability were subserved relatively independently by the two cerebral hemispheres. We performed this behavioral analysis in advance of obtaining neurophysiological results from non-human primates, which will ultimately provide insights on the neural mechanisms behind working memory resources. Unexpectedly, our results indicated that this was not the case. In fact, performance was generally higher for displays that involved stimuli appearing in the same (left or right) hemifield, in the spatial version of the working memory task. In cases where a subject exhibited a preference for stimuli on separate sides of the visual field, the advantage was not exclusive to the left- and right hemifield but it was present for lower and upper field as well, and it could be accounted for by the relative distance between stimuli. We failed to observe an independent-hemifield benefit in four different monkeys, performing the task at different levels of mastery, and appearing to rely on different strategies. No qualitative difference was observed between left/right and up/down fields, either, which would have suggested independence of working memory capacity based on hemispheric specialization, or for a shape working memory task.

There are a number of important limitations to our conclusions. Our results were obtained in variations of spatial-location and shape-working memory tasks, which represent a tiny fraction of tasks that can challenge the capacity of working memory. The stimulus displays we used are subject to grouping and Gestalt factors (discussed further below), which do not necessarily influence other types of visual working memory tasks. Additionally, we examined a small sample of non-human primates, recruited for the purposes of neurophysiological study, rather than behavioral analysis, *per se*. Nonetheless, our results provided clear examples of demanding working memory tasks that rely on visual working memory, and for which independent processing in the two hemispheres does not offer an advantage.

### Lateralization

Hemispheric specialization is well-known in humans for functions, such as language (localized predominantly in the left hemisphere) and visuo-spatial processing (in the right hemisphere). Less evidence of lateralization has been available in animal models, which do not possess language, though some traces of lateralization appear to be present in great apes (Corballis, 2012). Distributing stimuli between the two fields may therefore offer an advantage, at least for demanding tasks, which might benefit from the independent processing power of two hemispheres (Umemoto et al., 2010; Leblanc-Sirois and Braun, 2014). Indeed, imaging studies suggest that communication between hemispheres may increase with effort, during performance of complex tasks (Davis and Cabeza, 2015).

### Neural representation of multiple items in working memory

In order to understand the factors behind any potential lateralization, it is instructive to consider in more detail the neural mechanisms maintaining memory of multiple stimuli. Working memory is thought to be mediated by the persistent activity of neurons in the prefrontal cortex and other cortical areas, though alternative mechanisms have also been proposed in recent years (Stokes, 2015; Riley and Constantinidis, 2016). When multiple items are maintained in memory, then the activity of the subpopulation of neurons activated by each stimulus can be thought of as a “bump” (peak) in the network (Edin et al., 2009; Wimmer et al., 2014). Distributing the activity across separate populations of neurons, particularly in the two hemispheres, may in principle make the representation more robust, and less likely to be subject to interference, as is predicted by computational models (Compte et al., 2000). Indeed, neurophysiological studies examining correlates of working memory for multiple stimuli reveal that activity representing multiple stimuli degrades faster, and information saturates with the presentation of more than one stimulus, when these appear in the same, left or right hemifield, than when stimuli are presented bilaterally (Buschman et al., 2011; Matsushima and Tanaka, 2014). A contralateral representation of stimuli is also favored early after stimulus appearance, whereas bilateral representations appear at later responses (Kadohisa et al., 2015).

Other recent results, however, suggest that the organization of memory fields in the prefrontal cortex follows a quadrantic pattern of organization, with spatial working memory representations being biased by the vertical and horizontal meridians of the visual field, and functional connectivity between neurons more rarely crossing quadrant boundaries, rather than left vs. right field, specifically (Leavitt et al., 2017). Psychophysical studies in humans also confirm quadrant-level interference effects (Carlson et al., 2007). Our results are broadly consistent with these findings, as any behavioral effects we observed between left and right fields tended to be present between up and down fields, as well.

### Factors influencing working memory performance

There are other factors at work as well, that may counteract the benefits of distributing stimuli between hemispheres. There is strong evidence that the spatial relations of stimuli in a display play an important role in visual short term memory (Jiang et al., 2000). Spatial proximity (and other Gestalt principles, including connectedness, common region, and similarity) influence working memory performance (Peterson and Berryhill, 2013). Properties of nearby stimuli are better recalled from short-term memory (Xu, 2006). In that sense, stimuli appearing within the same hemifield and being subject to grouping principles may have an advantage over stimuli appearing at different hemifields.

Efficiencies may also be achieved by mentally transforming a multi-stimulus display, such as that used in our spatial working memory task into a polygon. In such an abstraction, higher performance may also be achieved when stimuli are nearby. In human studies, event-related potentials and BOLD fMRI activation elicited by visual displays amenable to grouping were diminished (i.e., require less resources for maintenance in memory) relative to displays of equal number of stimuli that cannot be grouped (Xu and Chun, 2007; Peterson et al., 2015). We should note however, that such an abstraction might be expected from animals that are able to master the task and strategically exploit stimulus grouping to perform it at a higher level. In our results, the greatest benefits of stimulus proximity were observed for animals that performed at the lowest levels (Figure 3).

We also saw no significant advantage of separating stimuli across the left and right hemifields in the shape working memory task, which does not lend itself to an obvious transformation or grouping process of this kind. On the other hand, we saw no benefit of grouping stimuli in this experiment, either. This finding may suggest that a potential advantage for same hemifield stimuli may only be present when no other task relevant stimulus features are available, although we should caution that our dataset was smaller in this experiment.

Taken together, our results argue that the proximity of stimuli maintained in memory provides advantages that counteract potential benefits of independent hemispheric processing, at least in the context of some working memory tasks.

## Author contributions

CC conceived and designed the research; HT and MR performed experiments; HT and CC analyzed data; HT and CC interpreted results of experiments; HT and CC drafted manuscript; HT, MR, and CC approved final version of manuscript.

### Conflict of interest statement

The authors declare that the research was conducted in the absence of any commercial or financial relationships that could be construed as a potential conflict of interest.
